# Comorbidities Associated with Granuloma Annulare: A Cross-Sectional, Case-Control Study

**DOI:** 10.3390/medicines7090053

**Published:** 2020-08-28

**Authors:** Erik Almazan, Youkyung S. Roh, Micah Belzberg, Caroline X. Qin, Kyle Williams, Justin Choi, Nishadh Sutaria, Benjamin Kaffenberger, Yevgeniy R. Semenov, Jihad Alhariri, Shawn G. Kwatra

**Affiliations:** 1 The Johns Hopkins University School of Medicine, Baltimore, MD 21205, USA; erikalmazan@jhmi.edu (E.A.); sophieroh@jhmi.edu (Y.S.R.); cqin8@jhmi.edu (C.X.Q.); kwill223@jhmi.edu (K.W.); jchoi222@uic.edu (J.C.); Nishadh.Sutaria@tufts.edu (N.S.); 2 Department of Dermatology, Johns Hopkins Hospital, Baltimore, MD 21287, USA; mbelzbe@jhu.edu; 3 Division of Dermatology, Ohio State University, Columbus, OH 43215, USA; Benjamin.Kaffenberger@osumc.edu; 4 Department of Dermatology, Massachusetts General Hospital, Boston, MA 02114, USA; YSEMENOV@mgh.harvard.edu

**Keywords:** granuloma annulare, granulomatous disorders of the skin, inflammatory skin conditions, medical dermatology

## Abstract

**Background:** Granuloma annulare (GA) is a cutaneous granulomatous disorder of unknown etiology. There are conflicting data on the association between GA and multiple systemic conditions. As a result, we aimed to clarify the reported associations between GA and systemic conditions. **Methods:** A retrospective, cross-sectional, case-control study was performed in which the medical records of biopsy-confirmed GA patients ≥18 years of age, who presented to the Johns Hopkins Hospital System between 1 January 2009 and 1 June 2019, were reviewed. GA patients were compared to controls matched for age, race, and sex. **Results:** After adjusting for confounders, GA patients (*n* = 82) had higher odds of concurrent type II diabetes (odds ratio (OR) = 5.27; 95% confidence interval (CI), 1.73–16.07; *p* < 0.01), non-migraine headache (OR = 8.70; 95% CI, 1.61–46.88; *p* = 0.01), and a positive smoking history (OR = 1.93; 95% CI, 1.10–3.38; *p* = 0.02) compared to controls (*n* = 164). Among GA patients, women were more likely to have ophthalmic conditions (*p* = 0.04), and men were more likely to have cardiovascular disease (*p* < 0.01) and type II diabetes (*p* = 0.05). No differences in systemic condition associations were observed among GA subtypes. **Conclusions:** Our results support the reported association between GA and type II diabetes. Furthermore, our findings indicate that GA may be associated with cigarette smoking and non-migraine headache disorders.

## 1. Introduction

Granuloma annulare (GA) is a granulomatous cutaneous disorder of unknown etiology with an estimated prevalence of 0.1–0.4% [1]. Clinically, GA has various presentations, including localized, generalized, subcutaneous, patch, or perforating subtypes [1,2,3,4]. Due to this variation in clinical presentation, characteristic histological findings are critical for diagnosis [2,4]. Given the unknown etiology of GA, studies have focused on uncovering its association with systemic conditions [2].

Systemic diseases proposed to have an association with GA include diabetes mellitus [1,5,6], dyslipidemia [7], hypothyroidism [8], and various malignancies [2]. However, results from the current literature have been conflicting, as several reports have also found no association between GA and diabetes mellitus [9], thyroid function [1], or dyslipidemia [5]. Additionally, reports on GA’s association with malignancy have largely been confined to case reports or studies with small sample sizes, which make an association difficult to ascertain [2]. Therefore, we performed a retrospective cross-sectional, case-control study of patients with clinically diagnosed and biopsy-confirmed GA to clarify the conflicting evidence of associations between GA and systemic conditions.

## 2. Materials and Methods

### 2.1. Study Design

Patients with GA were identified through a review of medical records at the Johns Hopkins Health System (JHHS), mainly comprised of tertiary-care, academic medical centers. A search was performed for patients with an ICD-10 (International Statistical Classification of Diseases and Related Health Problems, Tenth Revision) code L92.0 who received outpatient care between 1 January 2009 and 1 June 2019. Patients with incomplete medical records were excluded. The GA patient study cohort included patients 18 years or older with both a documented clinical presentation and biopsy consistent with GA, including histological evidence of lymphohistiocytic inflammation, mucin deposition, and collagen degradation. All clinical and histopathologic evaluations were performed by board-certified dermatologists and dermatopathologists at JHHS, respectively. Patients with incomplete medical records, lack of biopsy-confirmed GA, or foreign body reactions at the time of diagnosis were excluded. Patients with GA were matched to controls in a 1:2 ratio by age (±3 years), race, and sex. Controls presented to JHHS as outpatients for regularly scheduled skin exams or for benign, localized chief complaints. The study was found exempt by the JHHS institutional review board, and patient consent was waived as only de-identified data were used.

### 2.2. Data Collection

Histopathological reports and medical records of patients with GA were reviewed. Patient characteristics and comorbidities present on the date of GA diagnosis by pathology were manually extracted from medical records.

### 2.3. Definition of Smoking History and Comorbidities

Smoking history was defined as self-reported cigarette smoking at the time of GA diagnosis or a previous history of smoking, regardless of amount. Comorbidities were any diseases that were ongoing problems at the time of GA diagnosis. Cardiovascular disease included active problems such as atherosclerosis, arrhythmias, cardiomyopathies, and cardiac infections. A history of myocardial infarction, heart failure, and stroke was included even if they were past medical events, insofar as the sequelae of those events were deemed significant enough to be considered active problems in the patient medical record. Liver disease encompassed viral liver infections, non-alcoholic steatohepatitis, hepatic steatosis, autoimmune liver disease, and hereditary liver diseases. Ophthalmic conditions included retinal degeneration, vitreous degeneration, inflammatory conditions of the eye proper or the optic nerve, closed-angle glaucoma, open-angle glaucoma, cataracts, and myopia.

### 2.4. Statistical Analysis

Continuous variables were presented as mean ± standard deviation (SD), and categorical variables were analyzed as proportions. Means were compared between cohorts using Student’s *t*-test, while proportions were compared using the chi-squared or Fisher’s exact test, as appropriate. Logistic regression results were expressed using odds ratios with 95% confidence intervals. Analyses were conducted with Stata/SE, v. 15.1 (StataCorp LLC, College Station, TX, USA). Univariable analyses were performed to compare patient characteristics between the GA cohort and controls. Logistic regression was used to adjust for potential confounding variables in our comparisons. A *p*-value < 0.05 (two-tailed) was considered significant in all analyses.

## 3. Results

Patient billing codes and pathology reports identified 471 patients with an ICD-10 L92.0 code who were seen at JHHS from January 2009 to June 2019. A total of 82 patients (17.4%) met the inclusion criteria and were included in the retrospective chart review. Table 1 displays patient demographics and clinical characteristics. On average, both cohorts were aged 58 ± 16 years, predominately female, and of non-Hispanic white race. In regard to smoking, a greater proportion of GA patients had a history of smoking or were active smokers at the time of their diagnosis (*p* = 0.03). Type II diabetes mellitus (*p* < 0.01), liver disease (*p* = 0.04), and non-migraine headache (*p* = 0.02) were present more frequently in patients with GA compared to patients without GA. The prevalence of clinically diagnosed dyslipidemia (*p* = 0.41), hypothyroidism (*p* = 0.63), and solid organ malignancy (*p* = 0.76) did not significantly differ between the study groups.

Logistic regression was performed to address for potential confounding variables. A smoking history was associated with higher odds of GA (odds ratio (OR) = 1.93; 95% confidence interval (CI), 1.10–3.38; *p* = 0.02) after accounting for age, race, and sex. Patients with type II diabetes (*p* < 0.01), but not liver disease, also had higher odds of concurrent GA after accounting for age, race, sex, and smoking (Table 2). Non-migraine headache remained associated with GA after including age, race, sex, smoking history, cardiovascular disease, and essential hypertension in the logistic regression model (OR = 8.70; 95% CI, 1.61–46.88; *p* = 0.01).

A sub-analysis of patients with GA was performed, examining variation by sex (Table 3). Among GA patients, females were more likely than males to present with ophthalmic conditions (*p* = 0.04), while males were more likely than females to have concurrent cardiovascular disease (*p* < 0.01) and type II diabetes (*p* = 0.05). Further analysis by GA subtype, generalized or localized GA (Table 4), demonstrated that patients with localized GA were younger than patients with generalized GA (*p* < 0.01). Differences were not observed in comorbidities or smoking history by GA subtype.

## 4. Discussion

The results of our study investigating systemic disease associations with GA support previously published reports of an association between GA and type II diabetes. Additionally, our results align with previous studies which found no associations between GA and dyslipidemia [5], hypothyroidism [1], or malignancy [10,11]. However, we observed a significantly increased prevalence of a smoking history and non-migraine headaches among GA patients. An association between GA and a positive smoking history has not previously been reported, while associations with non-migraine headaches have not been reported outside of case reports.

Type II diabetes mellitus and dyslipidemia are two metabolic disorders that have been reported to be associated with GA. In 2009, a retrospective, multicenter study in Korea found a higher prevalence of diabetes mellitus in generalized GA patients compared to the general Korean population [6]. This observation was corroborated by similar findings in a retrospective analysis in which 44 GA patients in Taiwan were compared to the general Taiwanese population [5], as well as another study that found increased levels of fasting blood sugar in 28 Iranian GA patients compared to healthy controls [1]. However, another study using psoriasis patients as internal controls instead of national data failed to find a statistically significant association between GA and type II diabetes [9]. Studies exploring the relationship between GA and dyslipidemia have similarly observed conflicting results. A case-control study found significant associations between GA and dyslipidemia as well as increased levels of low-density lipoprotein, triglyceride, and total cholesterol [7]. However, these findings were not replicated in a more recent retrospective analysis in 2016 [5]. Our study found an association between type II diabetes and GA but did not observe a significant association between GA and dyslipidemia.

Hypothyroidism and solid organ malignancy are other systemic conditions with inconsistent reports on their associations with GA. A retrospective correlation study of 100 GA patients by Dabski and Winkelmann found 13 patients to have a thyroid disorder (in descending frequency: hypothyroidism, Grave’s disease, thyroiditis, thyroid adenoma) [12]. However, whether GA is truly associated with hypothyroidism is unclear. For example, while one small case-control study failed to demonstrate a significant difference in thyroid hormone levels between GA patients and healthy controls [1], another case-control study of similar sample size did, in fact, report an association between localized GA and autoimmune thyroiditis, specifically in adult women [8]. In the latter, the authors suggested a common immunogenetic pathophysiology potentially underlying the two conditions, as well as other systemic, GA-like, granulomatous conditions that lie on a spectrum [8]. Our study did not show that GA is associated with hypothyroidism. Collectively, these findings indicate that the current literature cannot definitely support or deny any association between GA and hypothyroidism. Additional large-scale, controlled studies are necessary to determine whether a true association between GA and hypothyroidism exists.

The question of whether or not malignancy is associated with GA is also debated. Multiple case reports have demonstrated GA occurring concurrently with various malignancies, which has led to speculation about a potential relationship [2]. While the exact etiology of GA is unknown, several studies have supported the hypothesis that the pathogenesis of GA involves a T-cell mediated response [13]. As a corollary, it has also been hypothesized that GA, in certain settings, may be a cutaneous manifestation of a chronic immune response to an underlying malignancy [14]. However, despite the seemingly high occurrence of GA in cases of malignancy, a meta-analysis of multiple case reports and correlation studies has not supported an association—with the caveat that clinically atypical GA in elderly individuals warrants investigation for an underlying malignancy that can histologically mimic GA [10]. A more recent review article by Hawryluk et al. echoed such conclusions, as well as cautioning against misdiagnoses, since other granulomatous dermatoses could in fact implicate malignancy [15]. Furthermore, a recent case-control study failed to find any association between malignancy and generalized GA [11]. Likewise, our study did not show that GA was associated with solid organ malignancy, thereby corroborating the results of the current literature on the topic.

The results of our study not only help to clarify previously reported associations between GA and certain systemic diseases but also reveal previously unrecognized relationships. For instance, we demonstrate increased prevalence of a smoking history in patients with GA. Even though the mechanism is unclear, smoking has been previously implicated in complications of sarcoidosis, another granulomatous disease [16]. Specifically, cigarette smoking has been shown to predict the development of ocular sarcoidosis in sarcoidosis patients. Smoking is thought to trigger systemic increases in cytokines, such as IL-6, IL-1β, and TNF-α, which are critical in the formation of granulomas [16]. As sarcoidosis and GA are both granulomatous diseases, it is possible that similar mechanisms may underlie the association between smoking and GA.

GA’s association with non-migraine headache has not been previously supported by a well-defined study. Our results showed that patients who received a diagnosis for GA were more likely to have non-migraine headache. Due to the lack of specific documentation in the medical record, it was not possible to identify the source of these non-migraine headaches. However, this finding is interesting due to reports of GA occurring simultaneously with giant cell arteritis (GCA), a particular cause of headaches. These conditions have been reported to occur together, and resolve with the same medications [17,18]. The presence of giant cells, granulomas, vascular deposition of IgM and C3, CD4 T-cell involvement [17], and an increased expression of HLA-B15 [18] in both GA and GCA lends credence to the possibility of a shared pathophysiologic mechanism. Despite the limitations of our study, it is possible that our non-migraine headache classification could have served as a proxy for a headache of a vascular origin. These findings encourage further inquiry to elucidate whether a relationship exists between specific headache disorders and GA, as well as their respective mechanisms.

Finally, our study found certain differences in the associations of systemic conditions with GA when stratified by sex. Females were more likely to present with a concurrent ophthalmic condition, while males were more likely to present with cardiovascular disease and type II diabetes. Given our sample size, it is difficult to determine whether these differences are generalizable. However, sex differences may be important to consider in future studies aiming to determine how GA presents in different patient populations.

This study had several limitations. Firstly, this was a retrospective study at an academic, tertiary-care medical center system, which may limit the generalizability of our results. Additionally, there was incomplete information in the medical record, which limited the conclusions that we could draw from our analyses. Important lab values and body mass indexes were difficult to obtain near the time of GA diagnosis. These values may have been confounding variables in our assessment of GA and comorbidities. Furthermore, the lack of detail in the medical record on the source of documented headaches did not allow for the assessment of whether or not GA was associated with a particular type of headache disorder. Lastly, our study was also limited by its sample size and study design. The limited GA cohort size affected the associations that could be made and parts of our cross-sectional study design do not allow us to determine causality.

A range of systemic conditions have been suggested to be associated with GA. Our study contributes to this literature by uncovering meaningful associations between GA and type II diabetes, a history of cigarette smoking, and non-migraine headache. In contrast to prior studies, no associations were detected between GA and dyslipidemia, hypothyroidism, and malignancy. These results contribute to the growing body of literature on GA and suggest further avenues for investigation.

## Figures and Tables

**Table 1:** Demographic and clinical characteristics of granuloma annulare (GA) patients.

Demographic and Clinical Characteristics	GA (*n* = 82)	Control (*n* = 164)	*p*-Value
Age (years), mean ± SD	58 ± 16	58 ± 16	0.99
Sex, n (%)			
Male	22 (27)	44 (27)	1.00
Female	60 (73)	120 (73)	
Race/Ethnicity, n (%)			
Non-Hispanic White	70 (85)	140 (85)	
Black	6 (7)	12 (7)	1.00
Asian	4 (5)	8 (5)	
Other	2 (2)	4 (2)	
Smoking History, n (%)	39 (48)	54 (33)	0.03
Comorbidities, n (%)			
Solid Organ Malignancy	3 (4)	8 (5)	0.76
HIV Positive Antibody	0 (0)	1 (1)	1.00
Ophthalmic Condition	26 (32)	56 (34)	0.70
Cardiovascular Disease	11 (13)	22 (13)	1.00
Unspecified Osteoarthritis	10 (12)	14 (9)	0.36
Depressive Disorder	6 (7)	15 (9)	0.63
Anxiety Disorder	6 (7)	12 (7)	1.00
Systemic Lupus Erythematosus	2 (2)	0 (0)	0.11
Type II Diabetes Mellitus	12 (15)	5 (3)	<0.01
Liver Disease	5 (6)	2 (1)	0.04
Dyslipidemia	24 (29)	40 (24)	0.41
Hypothyroidism	8 (10)	13 (8)	0.63
Essential Hypertension	21 (26)	44 (27)	0.84
Migraine	6 (7)	8 (5)	0.44
Non-Migraine Headache	6 (7)	2 (1)	0.02

**Table 2:** Logistic regression for comorbidities and granuloma annulare (GA) controlling for age, race, sex, and smoking.

Comorbidities (Yes/No)	Odds Ratio	95% Confidence Intervals	*p*-Value
Solid Organ Malignancy	0.64	0.16–2.59	0.53
HIV Positive Antibody	-	-	-
Ophthalmic Condition	0.84	0.46–1.53	0.57
Cardiovascular Disease	0.89	0.37–2.14	0.80
Unspecified Osteoarthritis	1.46	0.59–3.59	0.41
Depressive Disorder	0.68	0.25–1.87	0.46
Anxiety Disorder	0.91	0.32–2.60	0.86
Systemic Lupus Erythematosus	-	-	-
Type II Diabetes Mellitus	5.27	1.73–16.07	<0.01
Liver Disease	4.41	0.81–23.96	0.09
Dyslipidemia	1.24	0.66–2.34	0.50
Hypothyroidism	1.24	0.48–3.19	0.65
Essential Hypertension	0.84	0.44–1.62	0.61
Migraine	1.48	0.48–4.55	0.50
Non-Migraine Headache	7.55	1.45–39.30	0.02

**Table 3:** Demographic and clinical characteristics of granuloma annulare (GA) patients by sex.

Demographic and Clinical Characteristics	Female (*n* = 60)	Male (*n* = 22)	*p*-Value
Age (years), mean ± SD	57 ± 14	60 ± 19	0.50
Smoking History, n (%)	27 (45)	12 (55)	0.44
Comorbidities, n (%)			
Solid Organ Malignancy	2 (3)	1(5)	1.00
Ophthalmic Condition	23 (38)	3 (14)	0.04
Cardiovascular Disease	2 (3)	9 (41)	<0.01
Unspecified Osteoarthritis	6 (10)	4 (18)	0.45
Depressive Disorder	4 (7)	2 (9)	0.66
Anxiety Disorder	5 (8)	1 (5)	1.00
Systemic Lupus Erythematosus	2 (3)	0 (0)	1.00
Type II Diabetes Mellitus	6 (10)	6 (27)	0.05
Liver Disease	5 (8)	0 (0)	0.32
Dyslipidemia	16 (27)	8 (36)	0.42
Hypothyroidism	6 (10)	2 (9)	1.00
Essential Hypertension	13 (22)	8 (36)	0.25
Migraine	4 (7)	2 (9)	0.66
Non-Migraine Headache	6 (10)	0 (0)	0.18

**Table 4:** Demographic and clinical characteristics of granuloma annulare (GA) patients by subtype.

Demographic and Clinical Characteristics	Generalized (*n* = 47)	Localized (*n* = 35)	*p*-Value
Age (years), mean ± SD	63 ± 15	52 ± 14	<0.01
Smoking History, n (%)	23 (49)	16 (46)	0.77
Comorbidities, n (%)			
Solid Organ Malignancy	2 (4)	1(3)	1.00
Ophthalmic Condition	16 (34)	10 (29)	0.60
Cardiovascular Disease	5 (11)	6 (17)	0.39
Unspecified Osteoarthritis	6 (13)	4 (12)	1.00
Depressive Disorder	4 (9)	2 (6)	1.00
Anxiety Disorder	3 (6)	3 (9)	1.00
Systemic Lupus Erythematosus	1 (2)	1 (3)	1.00
Type II Diabetes Mellitus	6 (13)	6 (17)	0.58
Liver Disease	4 (9)	1 (3)	0.39
Dyslipidemia	14 (30)	10 (29)	0.90
Hypothyroidism	6 (13)	2 (6)	0.46
Essential Hypertension	11 (23)	10 (29)	0.60
Migraine	3 (6)	3 (9)	1.00
Non-Migraine Headache	3 (6)	3 (9)	1.00
